# Supplementary material for: Relationship between gut microbiota and circulating metabolites in population-based cohorts
Source: Nat Commun. 2019 Dec 20;10:5813. doi: 10.1038/s41467-019-13721-1 (PMC6925111; doi:10.1038/s41467-019-13721-1)
Supplement: Supplementary file 1 — Supplementary Information [file 41467_2019_13721_MOESM1_ESM.pdf]

Relationship between gut microbiota and circulating metabolites in population-based cohorts

Vojinovic *et al.*

Supplementary Information

Supplementary Figure 1. Plot of the effect estimate and 95% confidence interval for extremely-large, very-large, and some of the large VLDL lipoprotein subfractions generated in the analysis of gut microbiota and circulating metabolites while adjusting for age, sex, BMI, medication use, and multiple testing (n=2,309)…………………………………………………….……..……………3

Supplementary Figure 2. Plot of the effect estimate and 95% confidence interval for some of the large and medium VLDL lipoprotein subfractions generated in the analysis of gut microbiota and circulating metabolites while adjusting for age, sex, BMI, medication use, and multiple testing (n=2,309)…………………………………….…………………..………………..……………4

Supplementary Figure 3. Plot of the effect estimate and 95% confidence interval for medium VLDL lipoprotein subfractions generated in the analysis of gut microbiota and circulating metabolites while adjusting for age, sex, BMI, medication use, and multiple testing (n=2,309)…………….…………5

Supplementary Figure 4. Plot of the effect estimate and 95% confidence interval for small VLDL, IDL, LDL, and some of the very-large HDL lipoprotein subfractions generated in the analysis of gut microbiota and circulating metabolites while adjusting for age, sex, BMI, medication use and multiple testing (n=2,309)…………………………..………………………………….…………..……………6

Supplementary Figure 5. Plot of the effect estimate and 95% confidence interval for very-large, large, and medium HDL subfractions generated in the analysis of gut microbiota and circulating metabolites while adjusting for age, sex, BMI, medication use, and multiple testing (n=2,309)……………………....……………………………………..………..……………7

Supplementary Figure 6. Plot of the effect estimate and 95% confidence interval for small HDL subfractions, particle size, and some of the glycerides generated in the analysis of gut microbiota and circulating metabolites while adjusting for age, sex, BMI, medication use, and multiple testing (n=2,309)……………………………………...………………………………...……………8

Supplementary Figure 7. Plot of the effect estimate and 95% confidence interval for glycerides, cholesterol, fatty and amino acids, and acute-phase reaction markers generated in the analysis of gut microbiota and circulating metabolites while adjusting for age, sex, BMI, medication use, and multiple testing (n=2,309)…………………………………….……..…………….…………9

Supplementary Figure 8. Plot of the effect estimate and 95% confidence interval for very-large and large VLDL lipoprotein subfractions generated in the analysis of gut microbiota and circulating additionally adjusted for smoking and alcohol.…………………...…………………..…………..10

Supplementary Figure 9. Plot of the effect estimate and 95% confidence interval for some of the large and medium VLDL lipoprotein subfractions generated in the analysis of gut microbiota and circulating additionally adjusted for smoking and alcohol (n=2,309). …………………………....11

Supplementary Figure 10. Plot of the effect estimate and 95% confidence interval for some of the medium VLDL, small VLDL and very large HDL, IDL, and LDL lipoprotein subfractions generated

in the analysis of gut microbiota and circulating additionally adjusted for smoking and alcohol (n=2,309)...………………………………………………..……..…………………...………..…12

Supplementary Figure 11. Plot of the effect estimate and 95% confidence interval for very-large, large, and some of the small HDL lipoprotein subfractions generated in the analysis of gut microbiota and circulating additionally adjusted for smoking and alcohol (n=2,309). ……………....………..…13

Supplementary Figure 12. Plot of the effect estimate and 95% confidence interval for some of the small HDL lipoprotein subfractions, particle size, glycerides, cholesterol, fatty and amino acids, and acute-phase reaction markers generated in the analysis of gut microbiota and circulating additionally adjusted for smoking and alcohol (n=2,309). ……………………..…………………...…………..14

Supplementary Figure 13. Results of association analysis between metabolites and microbial genera and families assessed by linear regression analysis after adjustment for age, sex, body-mass index, technical covariates, medication use, smoking, and alcohol consumption (n=2,309)……..……..……15

Supplementary Figure 14. Venn diagram showing the number of microbial taxa associated with circulating triglycerides, very-low, low and high-density lipoprotein particles………..…………….16

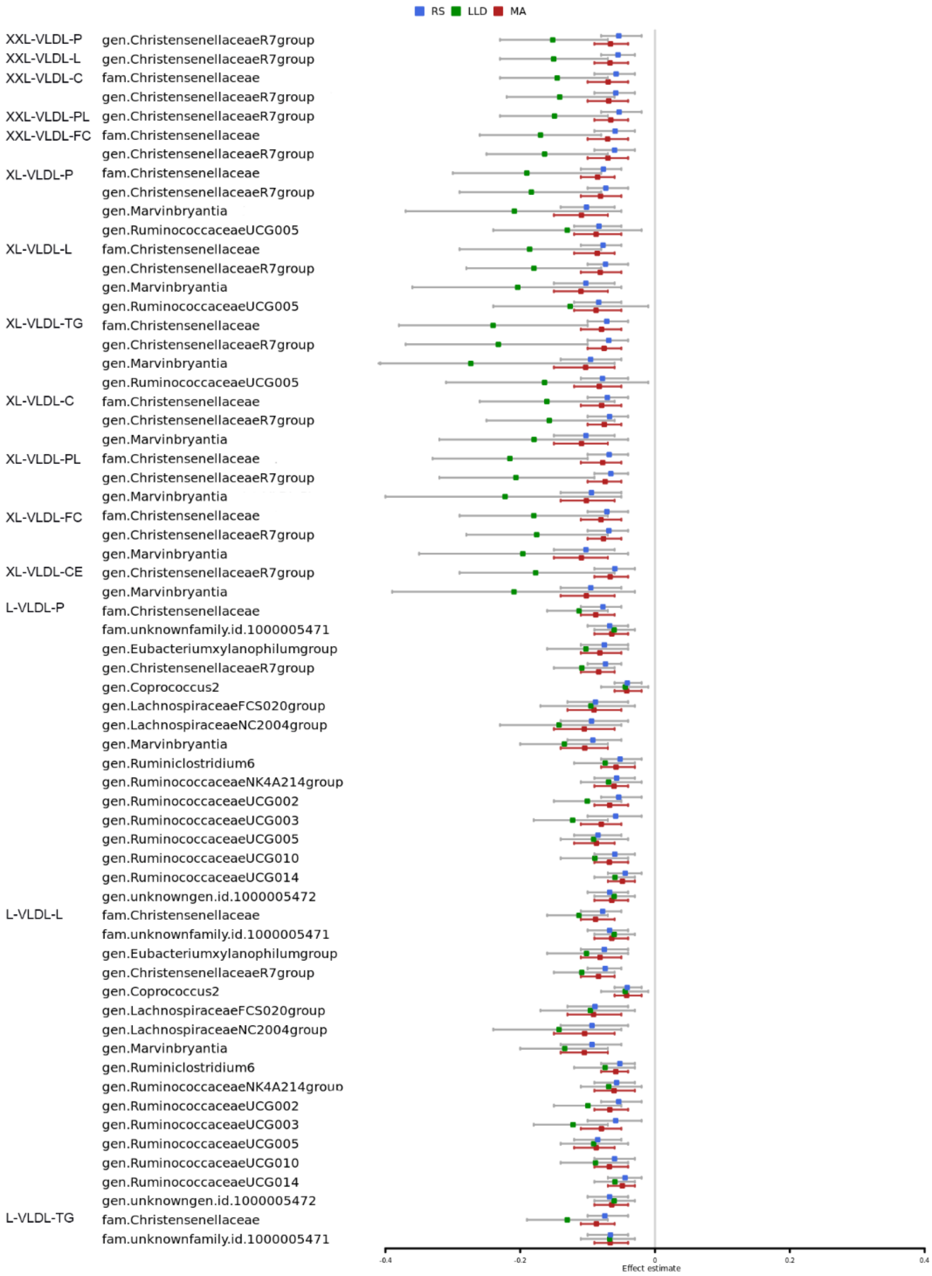

Supplementary Figure 1

Plot of the effect estimate and 95% confidence interval for extremely-large, very-large, and some of the large VLDL lipoprotein subfractions generated in the analysis of gut microbiota and circulating metabolites while adjusting for age, sex, BMI, medication use, and multiple testing (n=2,309). The results per cohort and combined summary statistic results are shown. Blue square denotes effect estimate in the Rotterdam Study, green in LifeLines-DEEP and red in meta-analysis. The solid vertical line represents a mean difference of 0 or no effect. Source data are provided as a Source Data file.

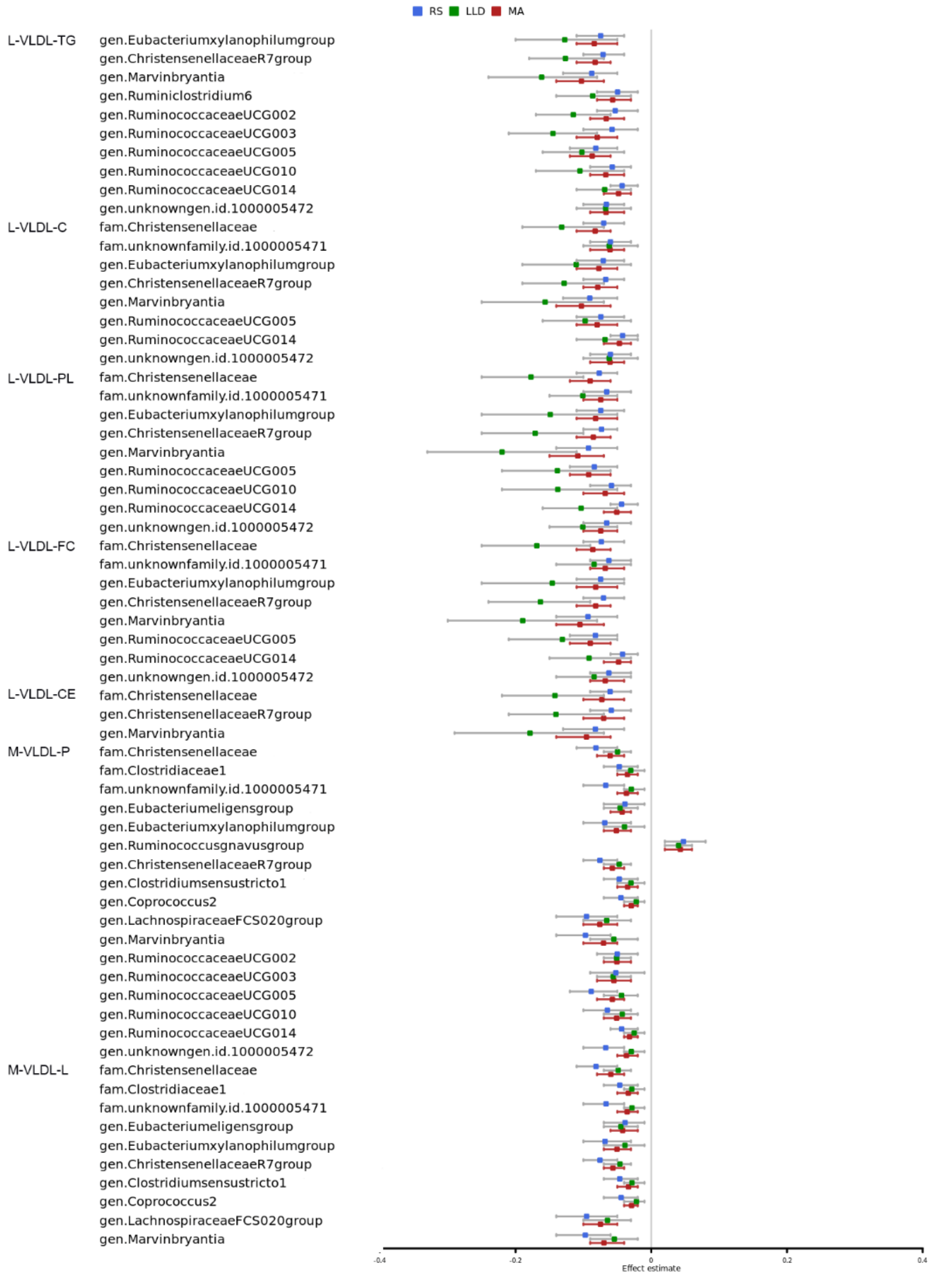

Supplementary Figure 2

Plot of the effect estimate and 95% confidence interval for some of the large and medium VLDL lipoprotein subfractions generated in the analysis of gut microbiota and circulating metabolites while adjusting for age, sex, BMI, medication use and multiple testing (n=2,309). The results per cohort and combined summary statistic results are shown. Blue square denotes effect estimate in the Rotterdam Study, green in LifeLines-DEEP and red in meta-analysis. The solid vertical line represents a mean difference of 0 or no effect. Source data are provided as a Source Data file.

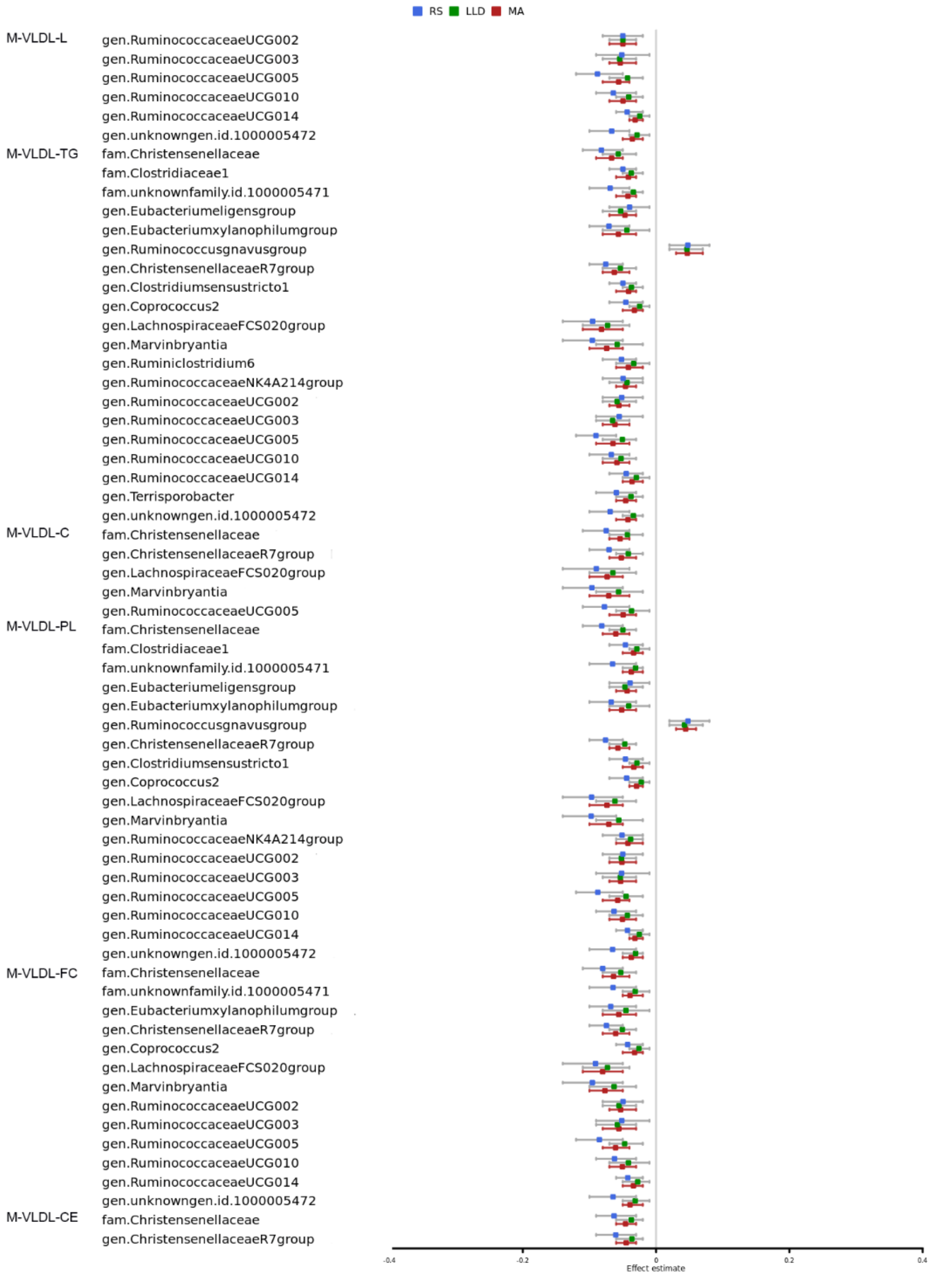

Supplementary Figure 3

Plot of the effect estimate and 95% confidence interval for medium VLDL lipoprotein subfractions generated in the analysis of gut microbiota and circulating metabolites while adjusting for age, sex, BMI, medication use and multiple testing (n=2,309). The results per cohort and combined summary statistic results are shown. Blue square denotes effect estimate in the Rotterdam Study, green in LifeLines-DEEP and red in meta-analysis. The solid vertical line represents a mean difference of 0 or no effect. Source data are provided as a Source Data file.

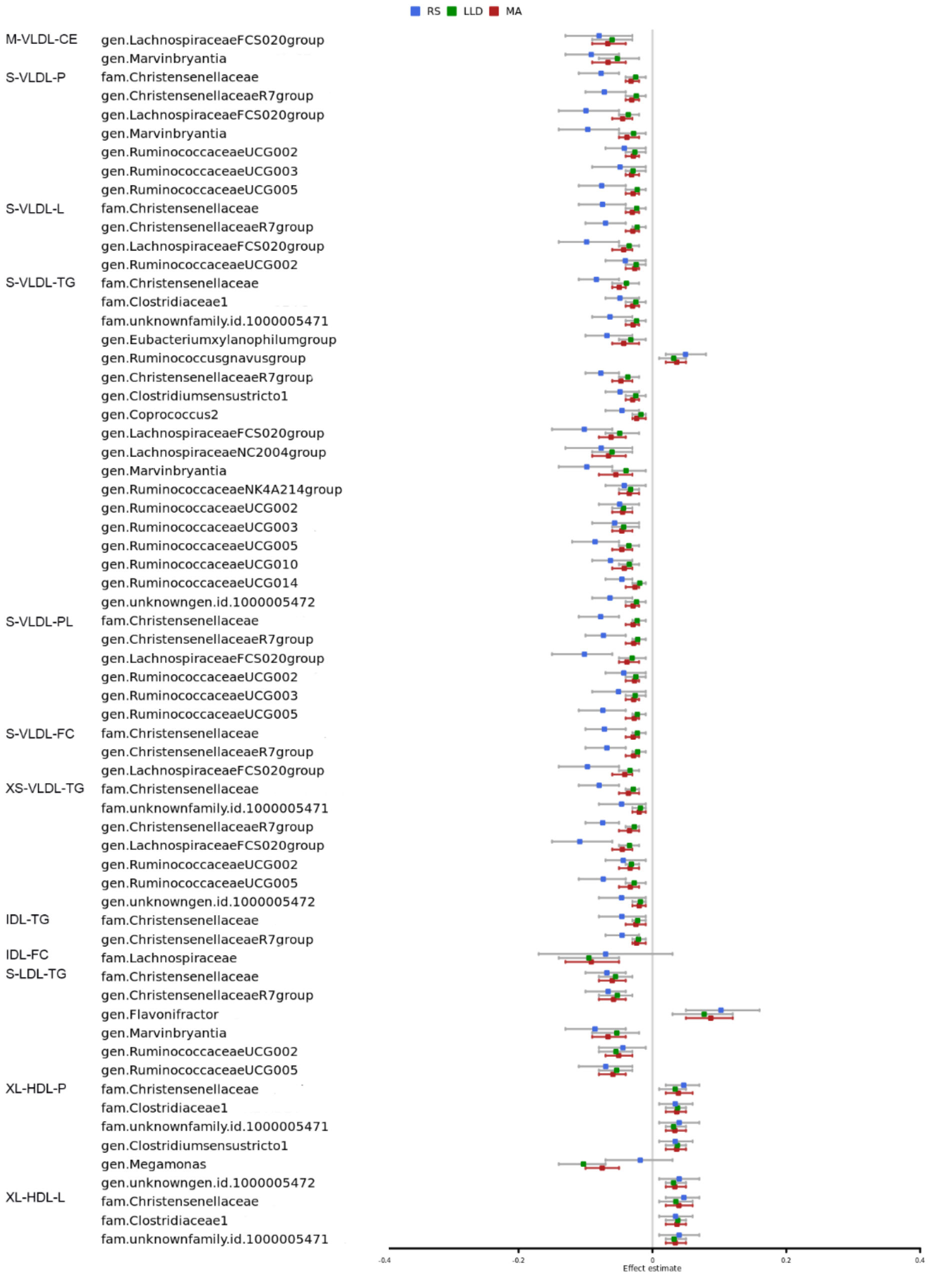

Supplementary Figure 4

Plot of the effect estimate and 95% confidence interval for small VLDL, IDL, LDL, and some of the very-large HDL lipoprotein subfractions generated in the analysis of gut microbiota and circulating metabolites while adjusting for age, sex, BMI, medication use and multiple testing (n=2,309). The results per cohort and combined summary statistic results are shown. Blue square denotes effect estimate in the Rotterdam Study, green in LifeLines-DEEP and red in meta-analysis. The solid vertical line represents a mean difference of 0 or no effect. Source data are provided as a Source Data file.

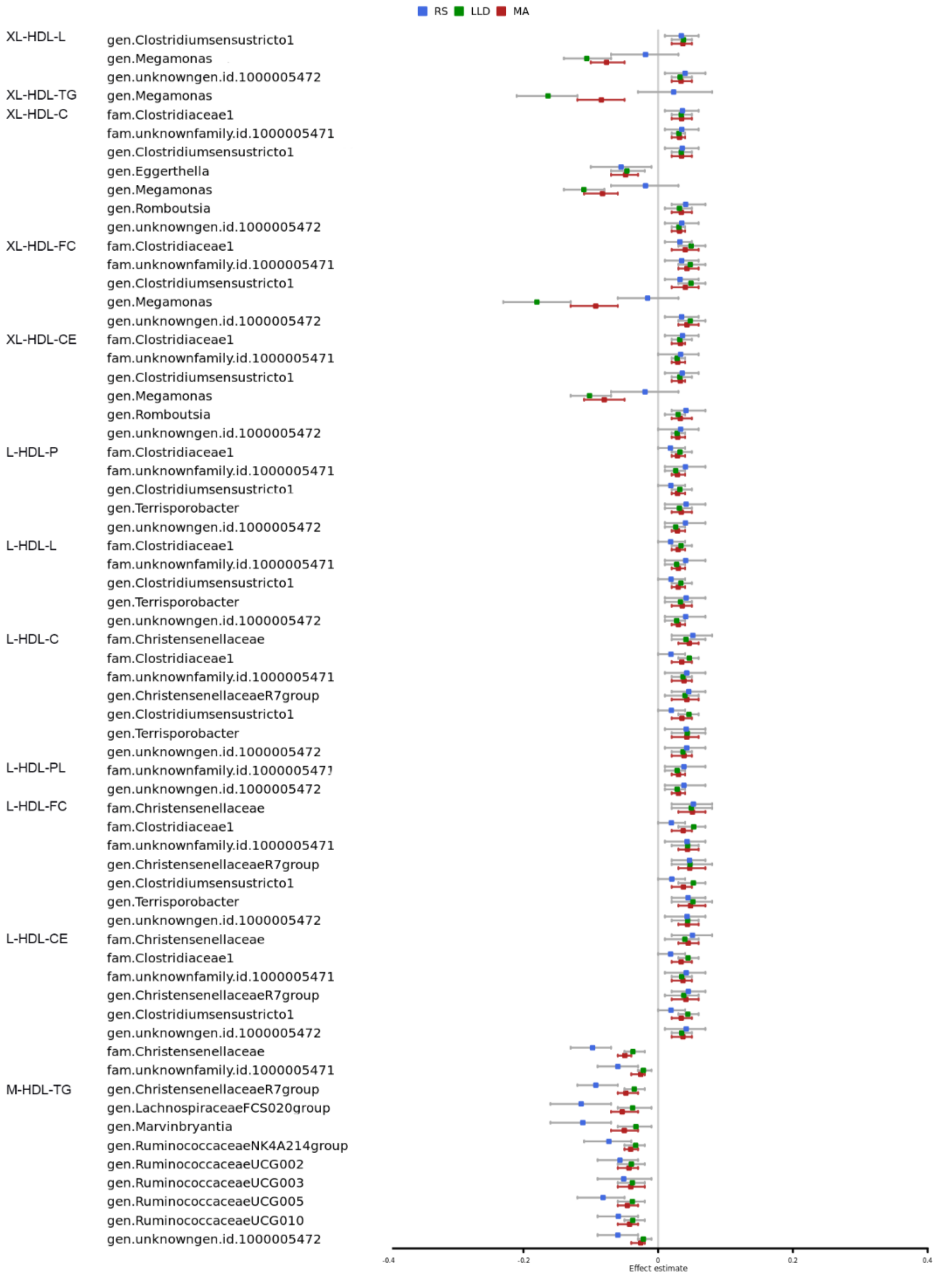

Supplementary Figure 5

Plot of the effect estimate and 95% confidence interval for very-large, large, and medium HDL subfractions generated in the analysis of gut microbiota and circulating metabolites while adjusting for age, sex, BMI, medication use and multiple testing (n=2,309). The results per cohort and combined summary statistic results are shown. Blue square denotes effect estimate in the Rotterdam Study, green in LifeLines-DEEP and red in meta-analysis. The solid vertical line represents a mean difference of 0 or no effect. Source data are provided as a Source Data file.

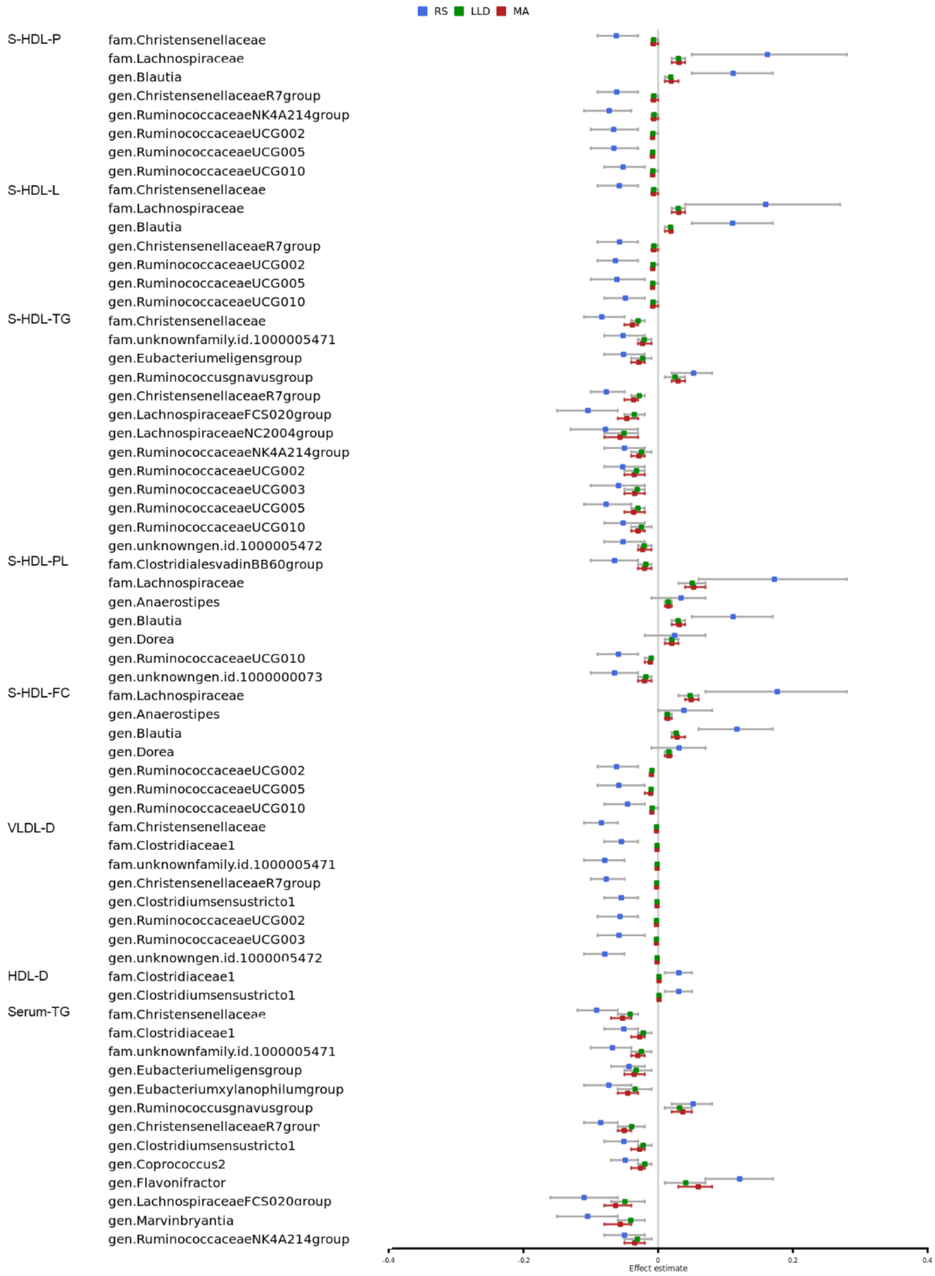

Supplementary Figure 6

Plot of the effect estimate and 95% confidence interval for small HDL subfractions, particle size, and some of the glycerides generated in the analysis of gut microbiota and circulating metabolites while adjusting for age, sex, BMI, medication use and multiple testing (n=2,309). The results per cohort and combined summary statistic results are shown. Blue square denotes effect estimate in the Rotterdam Study, green in LifeLines-DEEP and red in meta-analysis. The solid vertical line represents a mean difference of 0 or no effect. Source data are provided as a Source Data file.

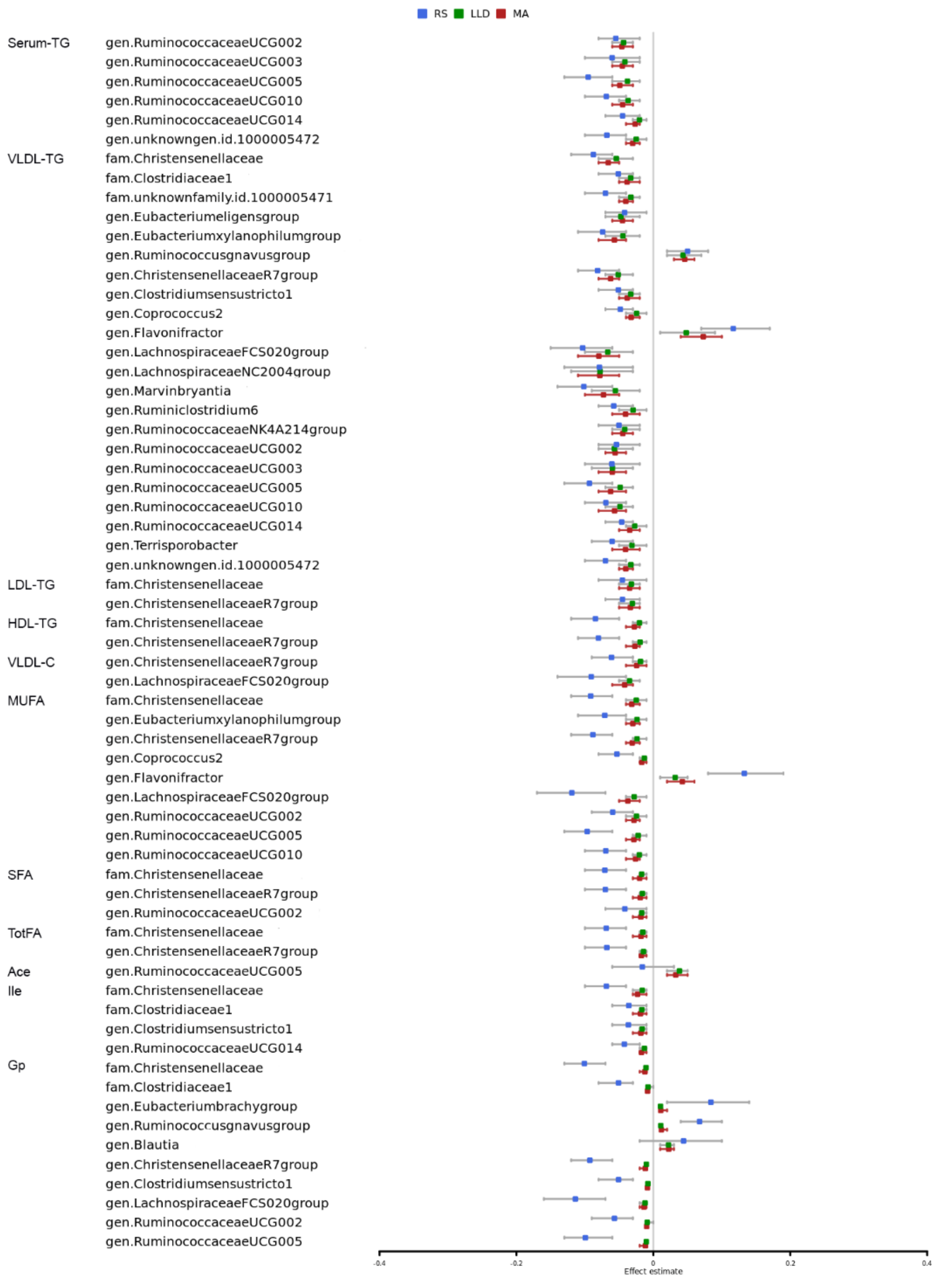

Supplementary Figure 7

Plot of the effect estimate and 95% confidence interval for glycerides, cholesterol, fatty and amino acids, and acute-phase reaction markers generated in the analysis of gut microbiota and circulating metabolites while adjusting for age, sex, BMI, medication use and multiple testing (n=2,309). The results per cohort and combined summary statistic results are shown. Blue square denotes effect estimate in the Rotterdam Study, green in LifeLines-DEEP and red in meta-analysis. The solid vertical line represents a mean difference of 0 or no effect. Source data are provided as a Source Data file.

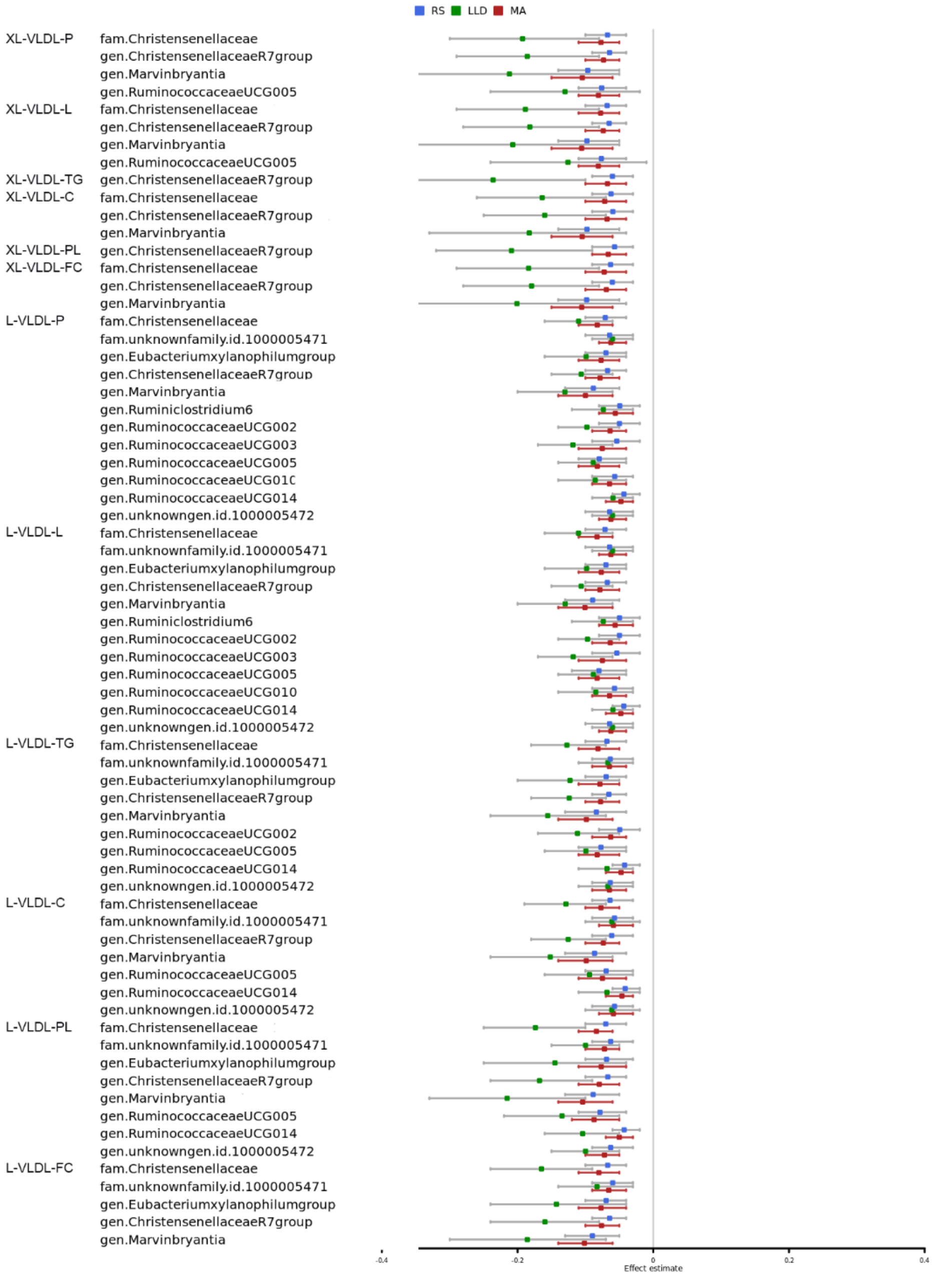

Supplementary Figure 8

Plot of the effect estimate and 95% confidence interval for very-large and large VLDL lipoprotein subfractions generated in the analysis of gut microbiota and circulating additionally adjusted for smoking and alcohol (n=2,309). The results per cohort and combined summary statistic results are shown. Blue square denotes effect estimate in the Rotterdam Study, green in LifeLines-DEEP and red in meta-analysis. The solid vertical line represents a mean difference of 0 or no effect. Source data are provided as a Source Data file.

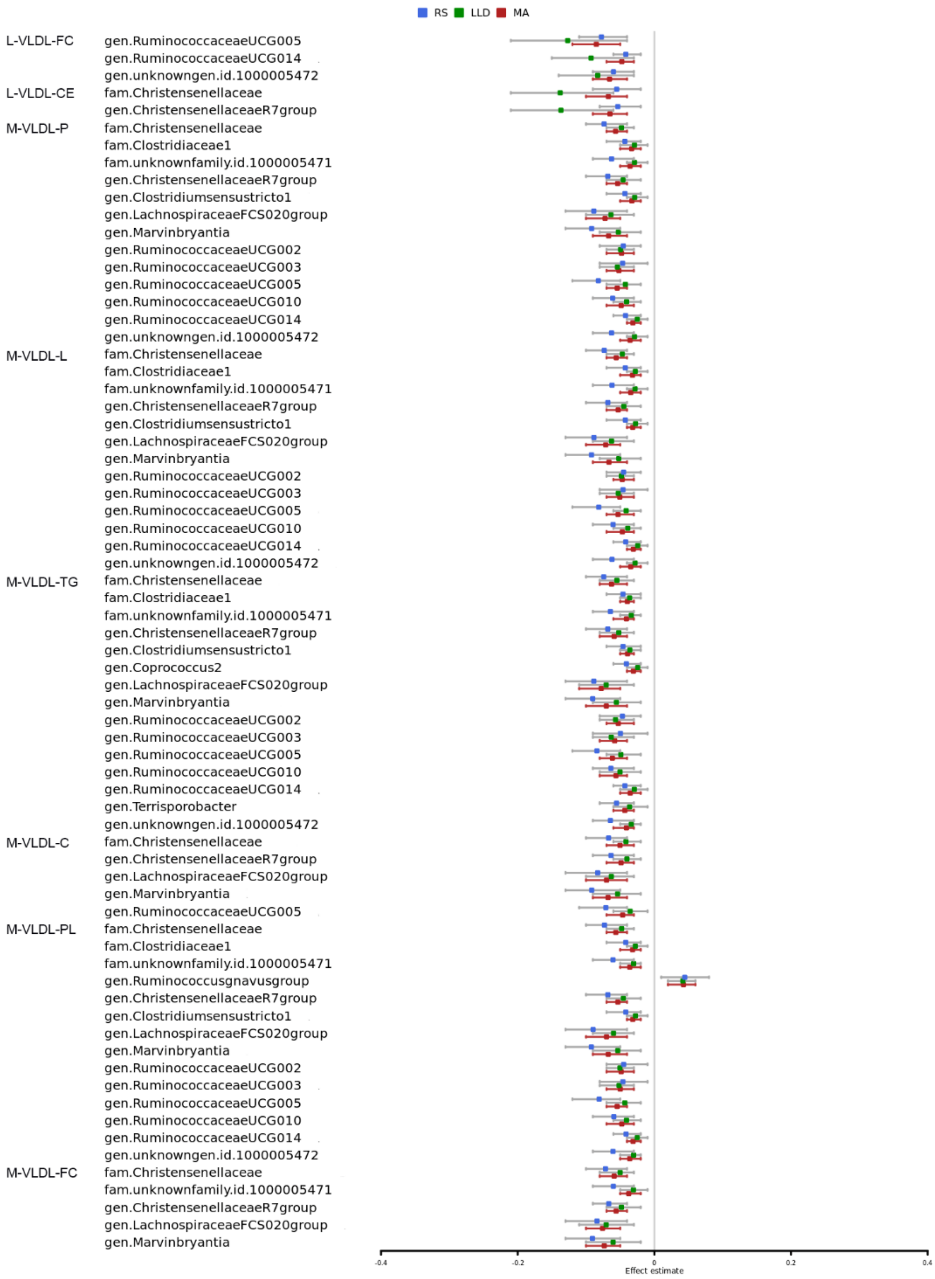

Supplementary Figure 9

Plot of the effect estimate and 95% confidence interval for some of the large and medium VLDL lipoprotein subfractions generated in the analysis of gut microbiota and circulating additionally adjusted for smoking and alcohol (n=2,309). The results per cohort and combined summary statistic results are shown. Blue square denotes effect estimate in the Rotterdam Study, green in LifeLines-DEEP and red in meta-analysis. The solid vertical line represents a mean difference of 0 or no effect. Source data are provided as a Source Data file.

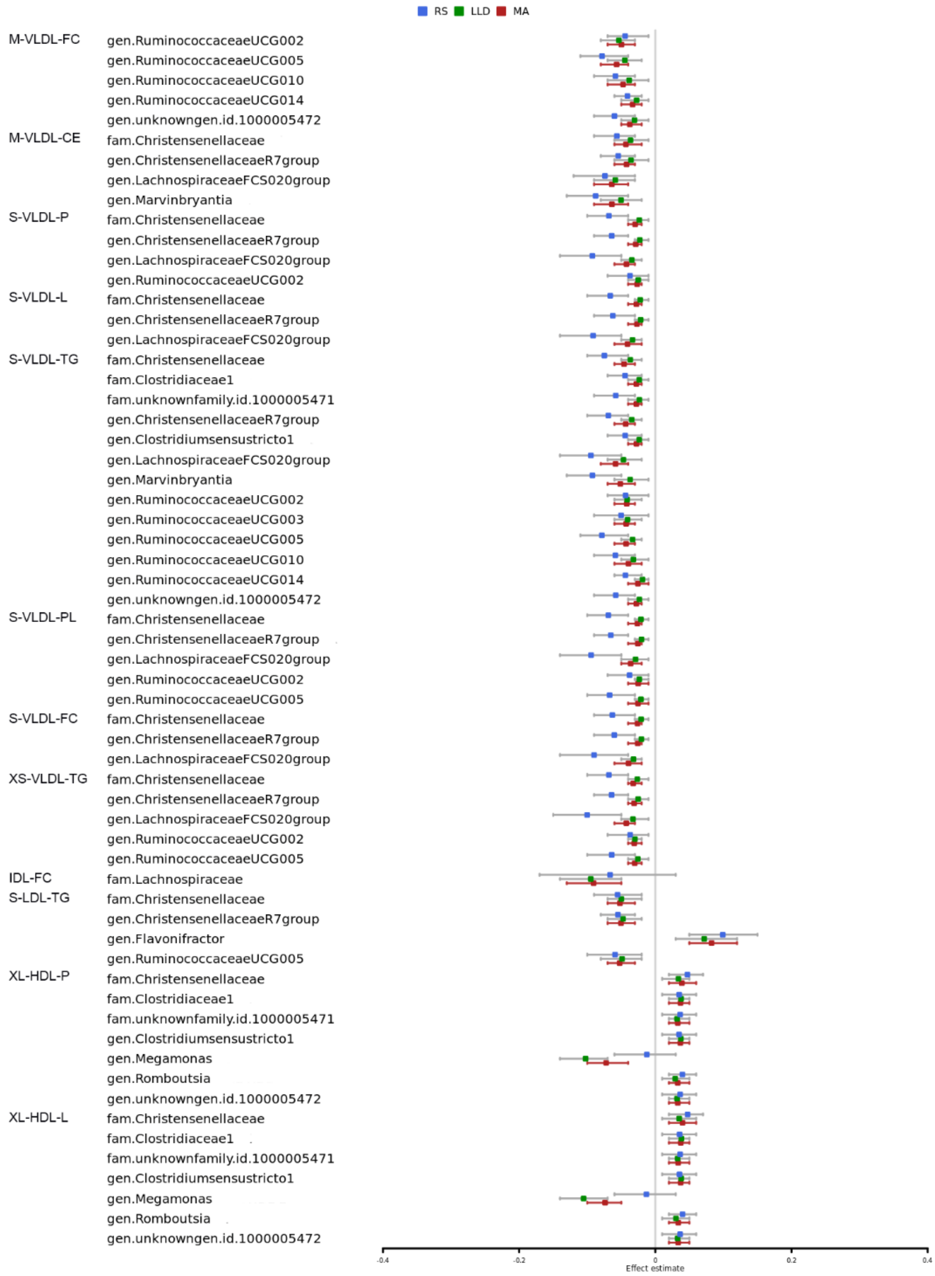

Supplementary Figure 10

Plot of the effect estimate and 95% confidence interval for some of the medium VLDL, small VLDL and very large HDL, IDL, and LDL lipoprotein subfractions generated in the analysis of gut microbiota and circulating additionally adjusted for smoking and alcohol (n=2,309). The results per cohort and combined summary statistic results are shown. Blue square denotes effect estimate in the Rotterdam Study, green in LifeLines-DEEP and red in meta-analysis. The solid vertical line represents a mean difference of 0 or no effect. Source data are provided as a Source Data file.

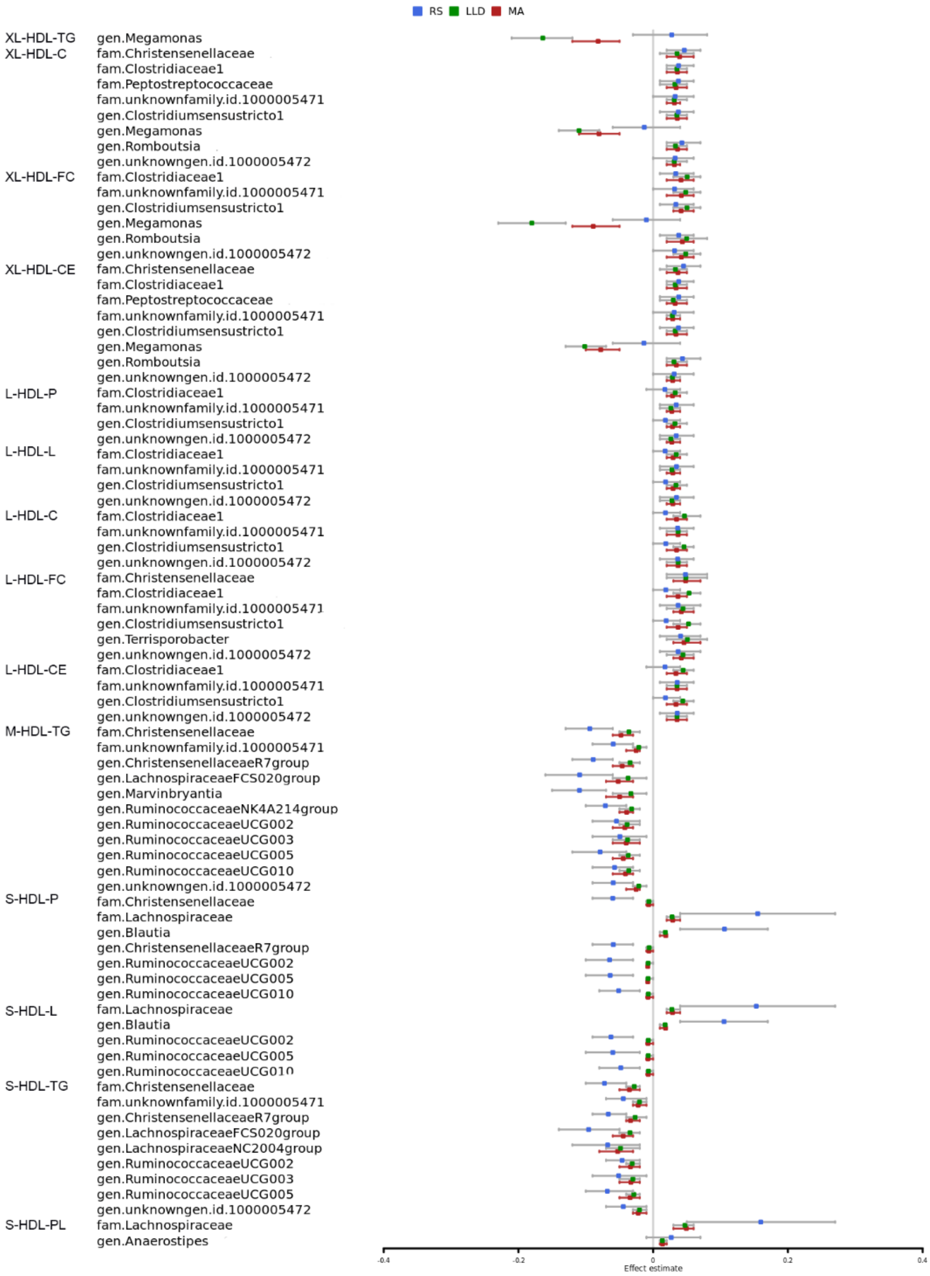

Supplementary Figure 11

Plot of the effect estimate and 95% confidence interval for very-large, large, and some of the small HDL lipoprotein subfractions generated in the analysis of gut microbiota and circulating additionally adjusted for smoking and alcohol (n=2,309). The results per cohort and combined summary statistic results are shown. Blue square denotes effect estimate in the Rotterdam Study, green in LifeLines-DEEP and red in meta-analysis. The solid vertical line represents a mean difference of 0 or no effect. Source data are provided as a Source Data file.

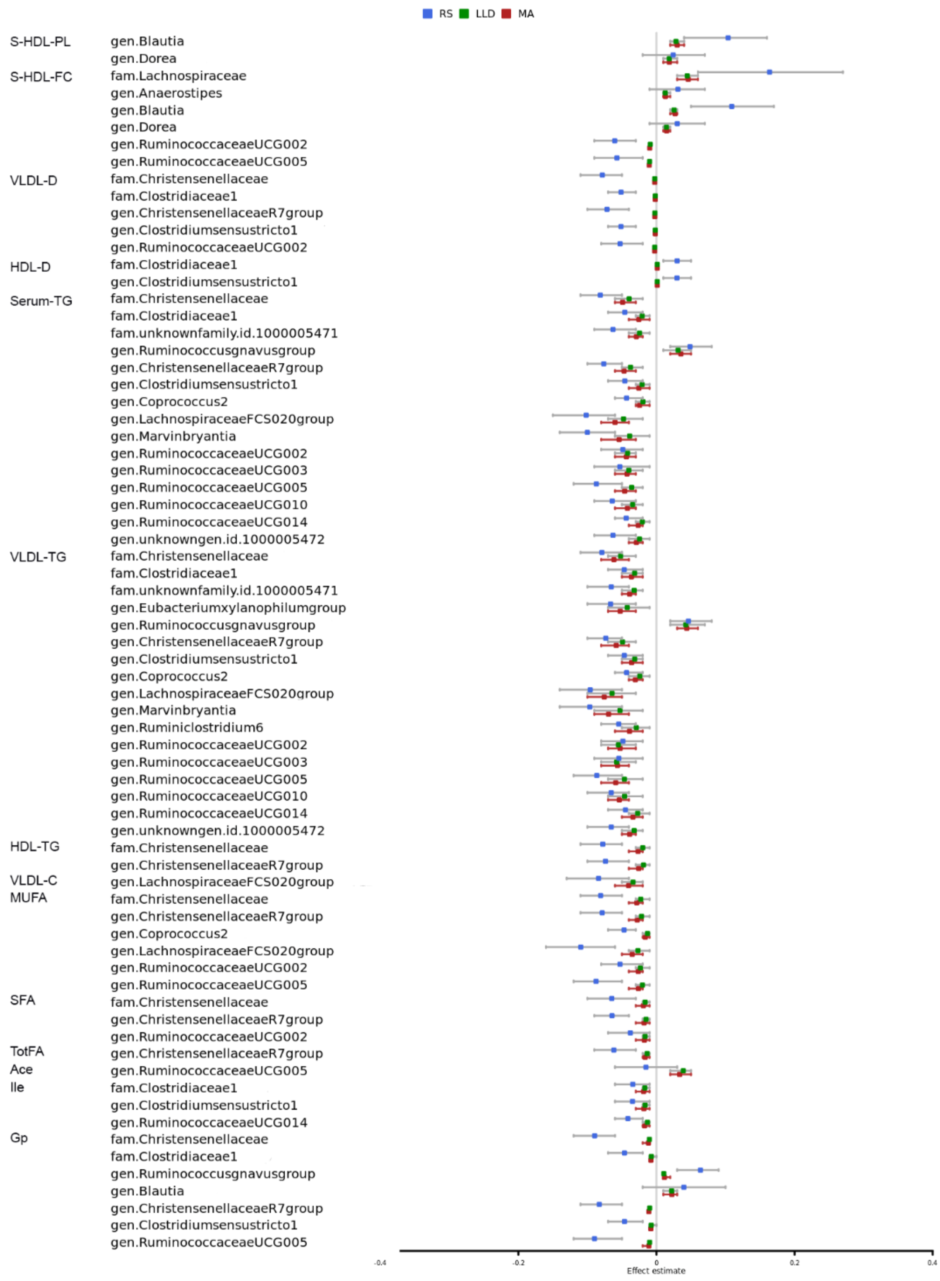

Supplementary Figure 12

Plot of the effect estimate and 95% confidence interval for some of the small HDL lipoprotein subfractions, particle size, glycerides, cholesterol, fatty and amino acids, and acute-phase reaction markers generated in the analysis of gut microbiota and circulating additionally adjusted for smoking and alcohol (n=2,309). The results per cohort and combined summary statistic results are shown. Blue square denotes effect estimate in the Rotterdam Study, green in LifeLines-DEEP and red in meta-analysis. The solid vertical line represents a mean difference of 0 or no effect. Source data are provided as a Source Data file.

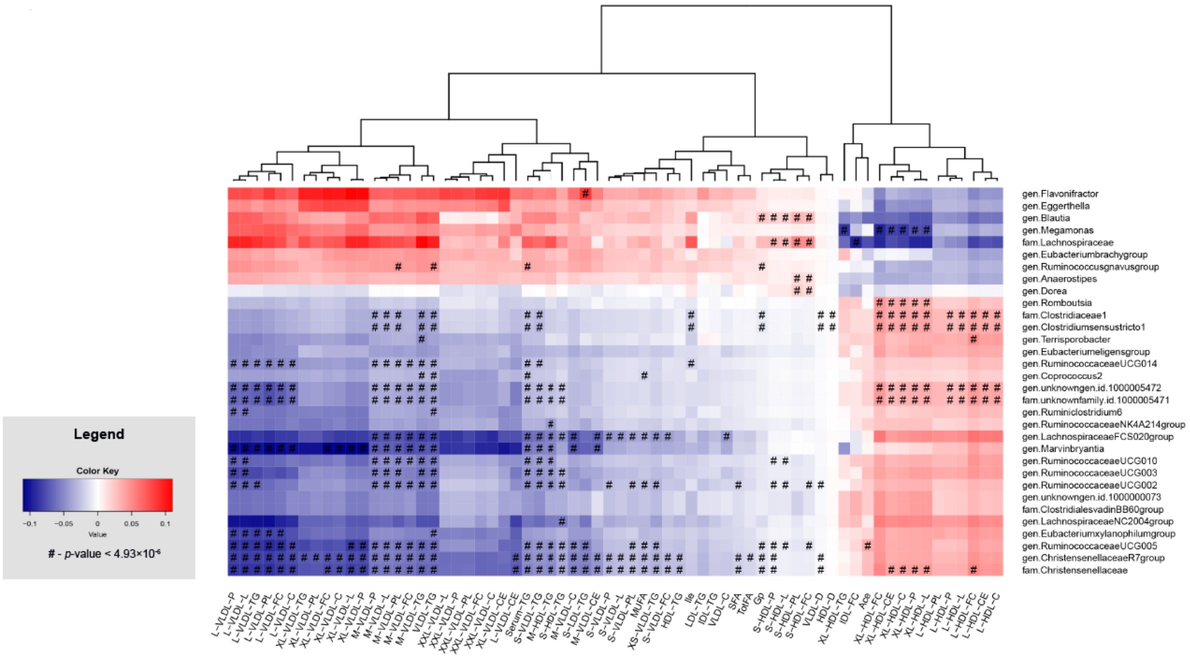

Supplementary Figure 13

Results of association analysis between metabolites and microbial genera and families assessed by linear regression analysis after adjustment for age, sex, body-mass index, technical covariates, medication use, smoking, and alcohol consumption (n=2,309). The hierarchical clustering of metabolites is used. Source data are provided as a Source Data file.

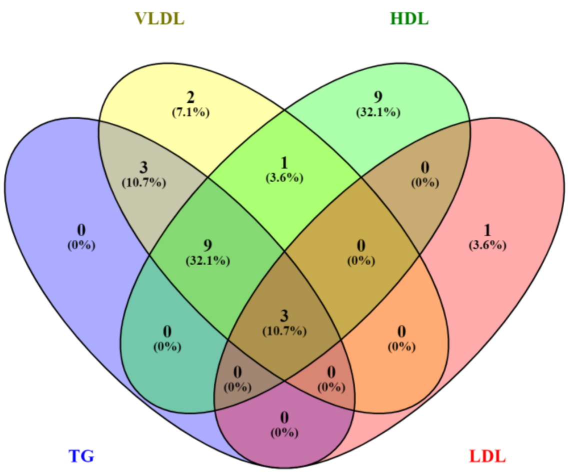

Supplementary Figure 14

Venn diagram showing the number of microbial taxa associated with circulating triglycerides, very-low, low and high-density lipoprotein particles.
