# Supplementary material for: Relationship between gut microbiota and circulating metabolites in population-based cohorts
Source: Nat Commun. 2019 Dec 20;10:5813. doi: 10.1038/s41467-019-13721-1 (PMC6925111; doi:10.1038/s41467-019-13721-1)
Supplement: Supplementary file 3 — Description of Additional Supplementary Files [file 41467_2019_13721_MOESM3_ESM.docx]

**Description of Additional Supplementary Files**

**File Name: Supplementary Data 1**

**Description:** List of all circulating metabolites tested for association with gut microbiota.

**File Name: Supplementary Data 2**

**Description:** List of microbial taxa tested for association with metabolites.

**File Name: Supplementary Data 3**

**Description:** Correlation matrix between all microbial taxa and metabolites.
