# Supplementary material for: Relationship between gut microbiota and circulating metabolites in population-based cohorts
Source: Nat Commun. 2019 Dec 20;10:5813. doi: 10.1038/s41467-019-13721-1 (PMC6925111; doi:10.1038/s41467-019-13721-1)
Supplement: Supplementary file 7 — Reporting Summary [file 41467_2019_13721_MOESM7_ESM.pdf]

nature research

Corresponding author(s): Cornelia van Duijn, Dina Vojinovic

Last updated by author(s): 5-11-2019

Reporting Summary

Nature Research wishes to improve the reproducibility of the work that we publish. This form provides structure for consistency and transparency in reporting. For further information on Nature Research policies, see Authors & Referees and the Editorial Policy Checklist .

Statistics

For all statistical analyses, confirm that the following items are present in the figure legend, table legend, main text, or Methods section.

n/a	Confirmed	
☐	☒	The exact sample size (*n*) for each experimental group/condition, given as a discrete number and unit of measurement
☐	☒	A statement on whether measurements were taken from distinct samples or whether the same sample was measured repeatedly
☐	☒	The statistical test(s) used AND whether they are one- or two-sided<br>*Only common tests should be described solely by name; describe more complex techniques in the Methods section.*
☐	☒	A description of all covariates tested
☐	☒	A description of any assumptions or corrections, such as tests of normality and adjustment for multiple comparisons
☐	☒	A full description of the statistical parameters including central tendency (e.g. means) or other basic estimates (e.g. regression coefficient) AND variation (e.g. standard deviation) or associated estimates of uncertainty (e.g. confidence intervals)
☐	☒	For null hypothesis testing, the test statistic (e.g. *F*, *t*, *r*) with confidence intervals, effect sizes, degrees of freedom and *P* value noted<br>*Give P values as exact values whenever suitable.*
☒	☐	For Bayesian analysis, information on the choice of priors and Markov chain Monte Carlo settings
☒	☐	For hierarchical and complex designs, identification of the appropriate level for tests and full reporting of outcomes
☐	☒	Estimates of effect sizes (e.g. Cohen's *d*, Pearson's *r*), indicating how they were calculated

Our web collection on statistics for biologists contains articles on many of the points above.

Software and code

Policy information about availability of computer code

Data collection	We have used RDP classifier (2.12) and SILVA 16S database (release 128) to reconstruct taxonomic composition of studied communities. Furthermore, we have used vegan package in R to calculate the microbial Shannon diversity index on taxonomic level of genera.
Data analysis	We have used rmeta package in R to perform inverse variance-weighted fixed-effect meta-analysis.

For manuscripts utilizing custom algorithms or software that are central to the research but not yet described in published literature, software must be made available to editors/reviewers. We strongly encourage code deposition in a community repository (e.g. GitHub). See the Nature Research guidelines for submitting code & software for further information.

Data

Policy information about availability of data

All manuscripts must include a data availability statement. This statement should provide the following information, where applicable:
- Accession codes, unique identifiers, or web links for publicly available datasets
- A list of figures that have associated raw data
- A description of any restrictions on data availability

All relevant data supporting the key findings of this study are available within the article and its supplementary information files. Data underlying Figure 1 and Supplementary Figures 1, 2 and 3 are provided as Source Data file. Other data are available from the corresponding author upon reasonable requests. Due to ethical and legal restrictions, individual-level data of the Rotterdam Study (RS) cannot be made publicly available. Data are available upon request to the data manager of the Rotterdam Study Frank van Rooij (f.vanrooij@erasmusmc.nl) and subject to local rules and regulations. This includes submitting a proposal to the management team of RS, where upon approval, analysis needs to be done on a local server with protected access, complying with GDPR regulations. The LifeLines-DEEP metagenomics sequencing data are available at the European Genome-phenome Archive under accession EGAS00001001704 [https://www.ebi.ac.uk/ega/studies/EGAS00001001704].

Field-specific reporting

Please select the one below that is the best fit for your research. If you are not sure, read the appropriate sections before making your selection.

☒ Life sciences ☐ Behavioural & social sciences ☐ Ecological, evolutionary & environmental sciences

For a reference copy of the document with all sections, see nature.com/documents/nr-reporting-summary-flat.pdf

Life sciences study design

All studies must disclose on these points even when the disclosure is negative.

Sample size	Our study population included 2,309 individuals from the Rotterdam Study and the LifeLines-DEEP cohort in whom metabolite measurements (Nightingale Health platform) and data on gut microbiota composition were assessed.
Data exclusions	Participants using antibiotics were excluded from the analysis.
Replication	In order to improve statistical power of the study, we combined the data of two large population-based studies. This also allowed us to internally cross-check consistency of the findings.
Randomization	Randomization is not needed for the type of study we preformed. Analysis were adjusted for age, sex, BMI, technical covariate, regular medication use, alcohol and smoking
Blinding	There was no group allocation during the data collection and/or analysis. The sample collection and statistical analysis were performed by different individuals.

Reporting for specific materials, systems and methods

We require information from authors about some types of materials, experimental systems and methods used in many studies. Here, indicate whether each material, system or method listed is relevant to your study. If you are not sure if a list item applies to your research, read the appropriate section before selecting a response.

Materials & experimental systems

n/a	Involved in the study	
☒	☐	Antibodies
☒	☐	Eukaryotic cell lines
☒	☐	Palaeontology
☒	☐	Animals and other organisms
☐	☒	Human research participants
☒	☐	Clinical data

Methods

n/a	Involved in the study	
☒	☐	ChIP-seq
☒	☐	Flow cytometry
☒	☐	MRI-based neuroimaging

Human research participants

Policy information about studies involving human research participants

Population characteristics	Mean age of 1,390 participants form Rotterdam Study included in this project was 56.9 years and 57.5 % were women. Mean age of 915 participants from LifeLines-DEEP was 44 years and 58,7% were women.
Recruitment	Population-based studies in the Netherlands with available gut microbiota composition dataset and circulating metabolites measured by Nuclear Magnetic Resonance technology (Nightingale Health platform).
Ethics oversight	Medical Ethics Committee of Erasmus Medical Center and board of the Netherlands Ministry of Health, Welfare and Sports approved Rotterdam Study, while Ethical Committee of the University Medical Center Groningen approved LifeLines-DEEP cohort.

Note that full information on the approval of the study protocol must also be provided in the manuscript.
